# *Neospora caninum* infection induced mitochondrial dysfunction in caprine endometrial epithelial cells via downregulating SIRT1

**DOI:** 10.1186/s13071-022-05406-4

**Published:** 2022-08-01

**Authors:** De-Liang Tao, Shan-Shan Zhao, Jin-Ming Chen, Xi Chen, Xin Yang, Jun-Ke Song, Qun Liu, Guang-Hui Zhao

**Affiliations:** 1 Department of Parasitology, College of Veterinary Medicine, Northwest A&F University, Yangling, 712100 China; 2 National Animal Protozoa Laboratory, College of Veterinary Medicine, China Agricultural University, Beijing, 100193 China

**Keywords:** *Neospora caninum*, Mitochondrial dysfunction, Caprine endometrial epithelial cells, Sirtuin 1, Autophagy, Propagation of parasite

## Abstract

**Background:**

Infection of *Neospora caninum*, an important obligate intracellular protozoan parasite, causes reproductive dysfunctions (e.g. abortions) in ruminants (e.g. cattle, sheep and goats), leading to serious economic losses of livestock worldwide, but the pathogenic mechanisms of *N. caninum* are poorly understood. Mitochondrial dysfunction has been reported to be closely associated with pathogenesis of many infectious diseases. However, the effect of *N. caninum* infection on the mitochondrial function of hosts remains unclear.

**Methods:**

The effects of *N. caninum* infection on mitochondrial dysfunction in caprine endometrial epithelial cells (EECs), including intracellular reactive oxygen species (ROS), mitochondrial membrane potential (MMP), adenosine triphosphate (ATP) contents, mitochondrial DNA (mtDNA) copy numbers and ultrastructure of mitochondria, were studied by using JC-1, DCFH-DA, ATP assay kits, quantitative real-time polymerase chain reaction (RT-qPCR) and transmission electron microscopy, respectively, and the regulatory roles of sirtuin 1 (SIRT1) on mitochondrial dysfunction, autophagy and *N. caninum* propagation in caprine EECs were investigated by using two drugs, namely resveratrol (an activator of SIRT1) and Ex 527 (an inhibitor of SIRT1).

**Results:**

The current study found that *N. caninum* infection induced mitochondrial dysfunction of caprine EECs, including accumulation of intracellular ROS, significant reductions of MMP, ATP contents, mtDNA copy numbers and damaged ultrastructure of mitochondria. Downregulated expression of SIRT1 was also detected in caprine EECs infected with *N. caninum*. Treatments using resveratrol and Ex 527 to caprine EECs showed that dysregulation of SIRT1 significantly reversed mitochondrial dysfunction of cells caused by *N. caninum* infection. Furthermore, using resveratrol and Ex 527, SIRT1 expression was found to be negatively associated with autophagy induced by *N. caninum* infection in caprine EECs, and the intracellular propagation of *N. caninum* tachyzoites in caprine EECs was negatively affected by SIRT1 expression.

**Conclusions:**

These results indicated that *N. caninum* infection induced mitochondrial dysfunction by downregulating SIRT1, and downregulation of SIRT1 promoted cell autophagy and intracellular proliferation of *N. caninum* tachyzoites in caprine EECs. The findings suggested a potential role of SIRT1 as a target to develop control strategies against *N. caninum* infection.

**Graphical abstract:**

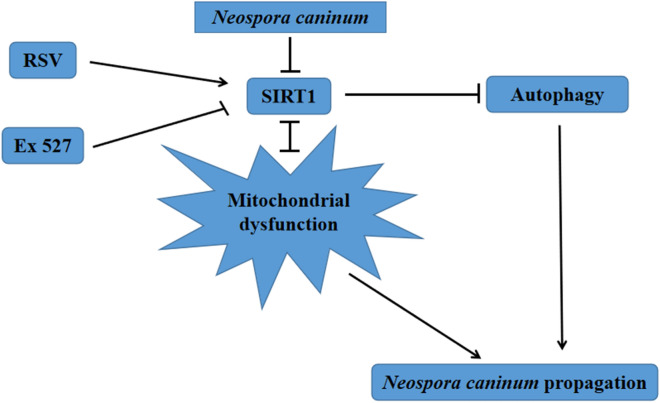

**Supplementary Information:**

The online version contains supplementary material available at 10.1186/s13071-022-05406-4.

## Background

Mitochondria, a functionally versatile organelle, are well appreciated as the powerhouse of cells to maintain energy production and stability by converting oxygen and gluconic metabolites into adenosine triphosphate (ATP) and to regulate redox signals [1, 2]. Recent evidence also indicated the pivotal role of mitochondria in modulating precise immune responses against infectious and sterile insults [3–6]. Mitochondrial dysfunction will occur when cells are subjected to a variety of adverse stimuli from external (e.g. infections of *Staphylococcus aureus*, bovine herpesvirus-1 and *Trypanosoma cruzi*) and internal (e.g. amyloid-β peptide and nitric oxide/peroxynitrite) sources, leading to abnormal mitochondrial morphology, reductions of the activities of mitochondrial oxidative phosphorylation complexes and ATP synthase, decreased mitochondrial membrane potential (MMP) and accumulation of reactive oxygen species (ROS) [7–11]. The aforementioned abnormalities could eventually cause oxidative stress, decreased energy supply, metabolic disorders, DNA damage, calcium dysregulation, cell apoptosis and inflammation [12–14]. Increasing data showed that mitochondrial dysfunction has been implicated in contribution to pathogenesis of neurodegeneration (e.g. Alzheimer’s disease, Parkinson’s disease), metabolic (e.g. diabetic kidney disease), cardiovascular (e.g. coronary artery disease, atherogenesis) and infectious (e.g. mastitis, hepatitis C, Chagas disease) diseases [15–20].

Neosporosis, caused by the obligate intracellular protozoan parasite *Neospora caninum*, is one of the main causes of abortion or stillbirths in pregnant cattle [21]. *Neospora caninum* infection has been reported to be responsible for approximately 12–42% of aborted fetuses in dairy cows and caused abortion in cattle with the median losses estimated as > US$ 1.298 billion per annum, with the highest to be US$ 2.380 billion [22, 23]. However, due to poor understanding of the pathogenic mechanisms of *N. caninum*, there are no effective drugs and vaccines available currently against this disease. *Neospora caninum* infection has been reported to induce accumulation of ROS and reduction of ATP levels, resulting in oxidative damage in cows and gerbils [24, 25]. Transcriptome analysis of cerebrovascular endothelial cells found that *N. caninum* infection induced increased expression of 21 mitochondrial genes that contributed to functions of Complex I, II, III, IV and V [26]. RNA-seq analysis of bovine trophoblast cells showed that *N. caninum* infection altered expression of several oxidoreductases (e.g. SOD2) [27]. However, few studies were conducted to unveil the mystery of mitochondrial dysfunction during *N. caninum* infection.

Sirtuin 1 (SIRT1), a NAD^+^-dependent histone deacetylase, has been reported to be an important regulator of metabolic control and mitochondrial biogenesis in a wide range of physiological processes and diseases (e.g. diabetes mellitus, aging and inflammatory diseases) and also identified as a probably promising therapeutic target to treat autoimmune diseases and reproductive failures [28–32]. Decreased expression of SIRT1 was found to be associated with mitochondrial dysfunction by increasing ROS and DNA damage in both male and female gametes [33]. The protective role of SIRT1 was also observed in several infectious diseases, including infections of viruses (e.g. respiratory syncytial virus and dengue virus), bacteria (e.g. *Pseudomonas aeruginosa* and *Helicobacter pylori*) and protozoan parasites (e.g. *Toxoplasma gondii*, *Trypanosoma cruzi* and *Cryptosporidium parvum*) [34–40]. In the current study, the mitochondrial dysfunction and its mechanisms associated with SIRT1 during *N. caninum* infection were investigated by using caprine endometrial epithelial cells (EECs).

## Materials and methods

### Cells, parasites and in vitro infection model

*Neospora caninum* Nc-1 wild-type strain was a gift from Prof. Qun Liu in China Agricultural University, Beijing, China. African green monkey kidney epithelial cells (Vero cells) used for passaging *N. caninum* tachyzoites were kindly provided by Prof. Xuefeng Qi from Northwest A&F University, Shaanxi, China. An in vitro model of *N. caninum* infection in caprine EECs (a gift from Prof. Yaping Jin in Northwest A&F University, Shaanxi, China) was established according to our previous study, with multiplicity of infection (MOI) of 3:1 (parasite:cell) [41].

### Determination of MMP

The MMP of intracellular mitochondria was monitored by using the mitochondrial membrane potential assay kit with JC-1 (Beyotime Biotechnology, Shanghai, China) according to the manufacturer's instructions. Briefly, caprine EECs were seeded in 12-well cell culture plates (Shanghai Sangon Biotech, Shanghai, China) and infected with *N. caninum* tachyzoites with a MOI of 3:1 (parasite:cell) for 48 h. Then, cells were washed with PBS and incubated with JC-1 (1 ×) in the dark for 30 min at 37 ℃. After washing with the JC-1 washing buffer (Beyotime Biotechnology, Shanghai, China), cells were observed under an inverted fluorescence microscopy (Leica Microsystems, Wetzlar, Germany) to detect fluorescence of green (excitation/emission wavelengths = 490/530 nm) and red (excitation/emission wavelengths = 525/590 nm). The relative MMP was expressed as the ratio of red/green fluorescence intensities.

### Determination of ROS

The intracellular ROS production was measured by using 2′,7′-dichloro-fluorescin diacetate (DCFH-DA) (Abmole, Shanghai, China). Caprine EECs were seeded in 12-well cell culture plates (Shanghai Sangon Biotech, Shanghai, China) and infected with *N. caninum* tachyzoites with a MOI of 3:1 (parasite:cell) for 48 h. The cells were washed with PBS and incubated with DCFH-DA (10 μM) in the dark for 20 min at 37 ℃. After washing the cells with serum-free DMEM/F12 medium for 5 min, the fluorescence intensity was detected under an inverted fluorescence microscopy (Leica Microsystems, Wetzlar, Germany). The fluorescence intensity of green (excitation/emission wavelengths of 488/530 nm) represented the intracellular ROS levels.

### Measurement of ATP levels

Intracellular ATP levels were measured by using an ATP Assay Kit (Beyotime Biotechnology, Shanghai, China) according to the manufacturer's instructions. Briefly, caprine EECs were seeded in six-well cell culture plates (Shanghai Sangon Biotech, Shanghai, China) and infected with *N. caninum* tachyzoites with a MOI of 3:1 (parasite:cell) for 48 h. The cells were lysed by using an ATP assay lysis solution, and then the cell lysis solution was incubated with an ATP assay working solution at room temperature for 2 min. The ATP content was measured in cells by utilizing a multifunctional fluorimeter microplate reader (Tecan Austria GmbH, Austria). The standard curve of ATP concentrations was prepared from known amounts (0.01, 0.03, 0.1, 0.3, 1, 3, 10, 30 μmol) of ATP levels. Results were expressed as arbitrary units of luminescence compared.

### Reverse transcriptase quantitative polymerase chain reaction (RT-qPCR)

Mitochondrial DNA (mtDNA) copy numbers were measured by using RT-qPCR with the templates of genomic DNA (gDNA) samples that were extracted using a Blood/Cell/Tissue DNA Extraction Kit (Tiangen, Beijing, China) according to the manufacturer's instructions. To determine the mRNA level for the *sirt1* gene during *N. caninum* infection, caprine EECs were seeded in 12-well cell culture plates (Shanghai Sangon Biotech, Shanghai, China) and infected with *N. caninum* tachyzoites with a MOI of 3:1 (parasite:cell) for 48 h. The cells were collected for RNA extraction with Trizol reagent (Accurate Biotechnology co., Ltd., Hunan, China). RNA samples were reverse transcribed to cDNA by using an *Evo M-MLV* RT Kit with gDNA Clean for RT-qPCR (Accurate Biotechnology Co., Ltd., Hunan, China). RT-qPCR reactions were performed by using 2 × Universal SYBR Green Fast RT-qPCR Mix (ABclonal, Wuhan, China) with specific primers listed in Additional file 1: Table S1. The 18 s rRNA gene was used to normalize the expression level of mtDNA (*nd1*), and the *glyceraldehyde phosphate dehydrogenase* (*gapdh*) gene was used to normalize the expression level of the *sirt1* gene. The relative expression of target genes was calculated by using the 2^−ΔΔCt^ method [42].

### Analysis of transmission electron microscopy (TEM)

Caprine EECs were seeded in six-well cell culture plates (Shanghai Sangon Biotech, Shanghai, China) and infected with *N. caninum* tachyzoites with a MOI of 3:1 (parasite:cell) for 48 h. The cells (about 10^7^ cells per sample) were washed with PBS, digested with trypsin and collected by centrifuge at 1000 rpm for 5 min. Cell pellets were incubated with 2.5% glutaraldehyde overnight at 4 ℃ and postfixed with 1% osmium tetroxide for 2–3 h. Fixed cells were dehydrated with increasing concentrations of ethanol, infiltrated with resin and embedded. Ultrathin sections were obtained by using an ultramicrotome (Leica Microsystems, Wetzlar, Germany), double stained with 4% uranyl acetate and lead citrate and analyzed by using a transmission electron microscopy (Hitachi Ltd., Tokyo, Japan).

### Western blot analysis

Caprine EECs were seeded in six-well cell culture plates (Shanghai Sangon Biotech, Shanghai, China) and infected with *N. caninum* tachyzoites with a MOI of 3:1 (parasite:cell) for 48 h. The cells were collected for protein extraction by using a RIPA lysis buffer (Beijing Applygen Technologies Co., Ltd., Beijing, China) with 1 mM PMSF and protein inhibitors (Beijing Solarbio Science & Technology Co., Ltd., Beijing, China). Protein concentrations were measured using a BCA assay kit (Beyotime Biotechnology, Shanghai, China). A total of 25 μg of proteins was separated by 10% (for SIRT1) or 12% (for sequestosome 1, SQSTM1/p62) or 15% (for microtubule-associated protein light chain, LC-3II) polyacrylamide gel electrophoresis and transferred to PVDF membranes (Millipore, Billerica, MA, USA). The membranes were blocked with 5% nonfat milk powder (Shanghai Sangon Biotech, Shanghai, China) for 2 h and incubated overnight with antibodies against SIRT1 (1:2000, Abways, Shanghai, China), p62 (1:5000, Abways, Shanghai, China), LC-3II (1:5000, Abways, Shanghai, China) and β-actin (1:5000, ABclonal, Wuhan, China) at 4 °C. Horseradish peroxidase (HRP)-conjugated donkey anti-rabbit antibody (1:5000, Abclonal, Wuhan, China) was used as the secondary antibody for all reactions and incubated with antibodies above at room temperature for 1 h. The images were visualized using an enhanced chemiluminescence (ECL) system (Beijing Applygen Technologies Co., Ltd., Beijing, China), and densitometry analysis of interest protein bands was calculated using the ImageJ software (https://imagej.net/Fiji/Downloads).

### Drug treatment and propagation of *N. caninum*

To investigate the effect of SIRT1 on propagation of *N. caninum*, caprine EECs were incubated with an activator resveratrol (RSV) (Beijing Solarbio Science & Technology Co., Ltd., Beijing, China) or an inhibitor Ex 527 (Beyotime Biotechnology, Shanghai, China) of SIRT1 for 1 h and then infected with *N. caninum* tachyzoites at a MOI of 3:1 (parasite:cell) for 48 h. The numbers of *N. caninum* tachyzoites per parasitophorous vacuole were calculated by using inverted optical microscopy (Olympus Co., Tokyo, Japan), and a total of 100 vacuoles were counted. In addition, the cytotoxicities of RSV and Ex 527 were analyzed using a cell counting kit (CCK-8; Zeta life, California, USA), and both 50 μM RSV and 20 μM Ex 527 had no significant cytotoxicities for caprine EECs (Additional file 2: Fig. S1).

### Statistical analysis

Data were reported as means ± standard deviation (SD) in at least three independent experiments, and the differences between independently experimental data were analyzed using GraphPad PRISM 6.07 (GraphPad Software Inc., San Diego, CA, USA). *P* values were computed using the two-tailed *t* test, with a parametric test. *P* value < 0.05 (*P < 0.05; **P < 0.01; ***P < 0.001) compared with an appropriate control group was considered as statistically significant.

## Results

### Occurrence of mitochondrial dysfunction in caprine EECs induced by *N. caninum* infection

**Fig. 1:**
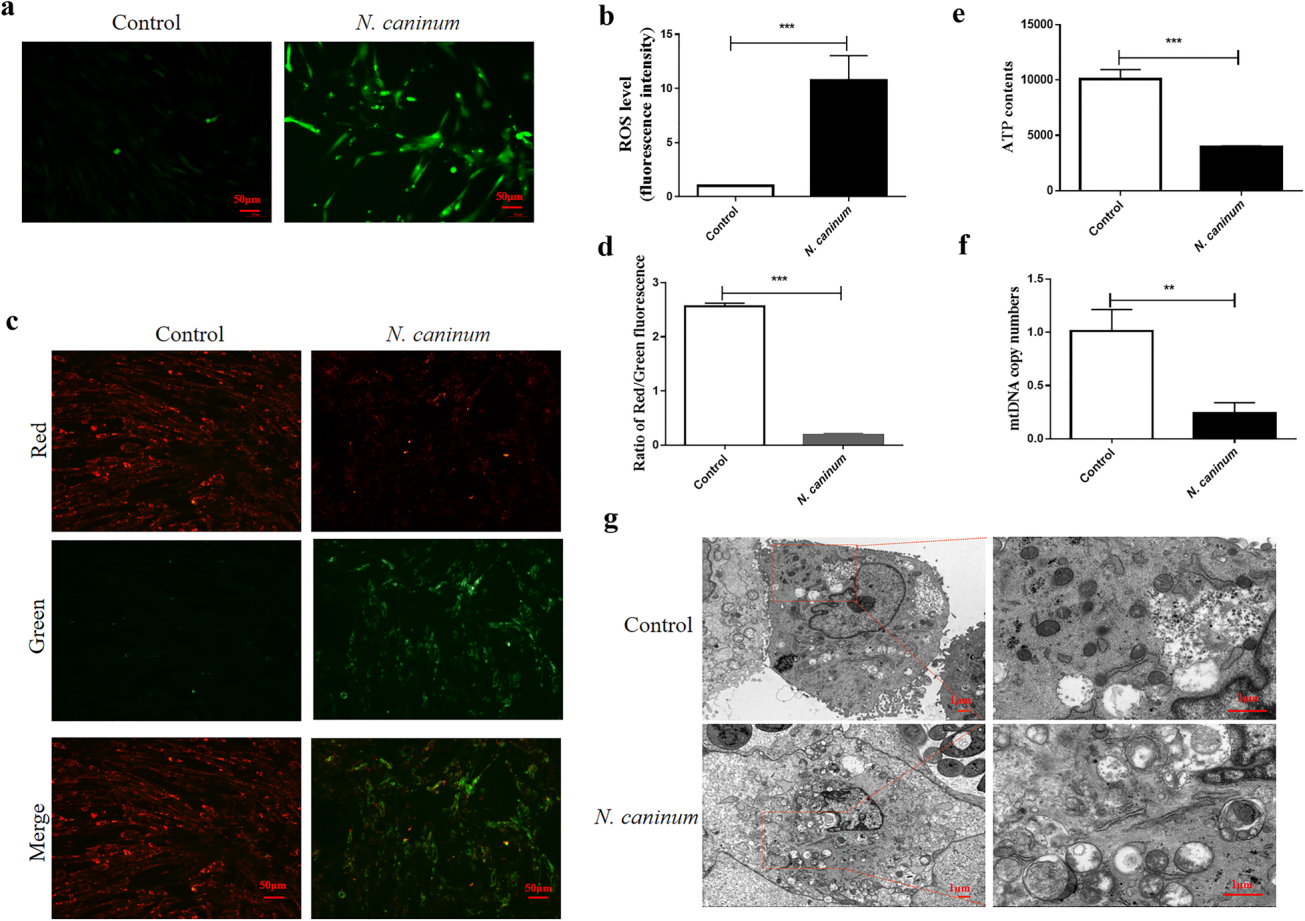
Mitochondrial dysfunction in caprine endometrial epithelial cells (EECs) induced by *Neospora caninum* infection. **a**, **b** ROS levels investigated by a DCFH-DA fluorescent probe in caprine EECs infected with *N. caninum* tachyzoites at a multiplicity of infection (MOI) of 3:1 (parasite:cell) for 48 h. Scale bar, 50 µm. **c**, **d** The mitochondrial membrane potential (MMP) detected by JC-1 in caprine EECs at 48 h post infection. Scale bar, 50 µm. **e** ATP contents detected by an ATP Determination Kit in caprine EECs at 48 h. **f** Mitochondrial DNA (mtDNA) copy numbers detected by qPCR in caprine EECs at 48 h. **g** Mitochondrial ultrastructure morphology in caprine EECs detected at 48 h by using transmission electron microscopy (TEM). Scale bar, 1 µm. Data are shown as mean ± standard deviation (SD) of three independent experiments. *P*-values were calculated using Student’s *t* test. **P < 0.01; ***P < 0.001

To evaluate the mitochondrial function during *N. caninum* infection, the ROS levels, MMP, ATP levels and mtDNA copy numbers were determined in caprine EECs infected with *N. caninum* for 48 h (Fig. 1). Compared to the control group without infection, *N. caninum* infection induced a significant increase of ROS production in caprine EECs (Fig. 1a, b). The ratios of red/green fluorescence intensities were significantly decreased in infected caprine EECs, indicating reduction of the relative MMP induced by *N. caninum* infection (Fig. 1c, d). Significant reductions were also detected for ATP levels (Fig. 1e) and mtDNA copy numbers (Fig. 1f) in infected caprine EECs. Furthermore, mitochondrial ultrastructural changes, e.g. cristae fractures, mitochondrial deformation, swelling, and vacuolization and mitochondrial autophagy, were observed by using TEM (Fig. 1g). These results showed occurrence of mitochondrial dysfunction in caprine EECs induced by *N. caninum* infection*.*

### Effect of SIRT1 on mitochondrial dysfunction in caprine EECs induced by *N. caninum* infection

**Fig. 2:**
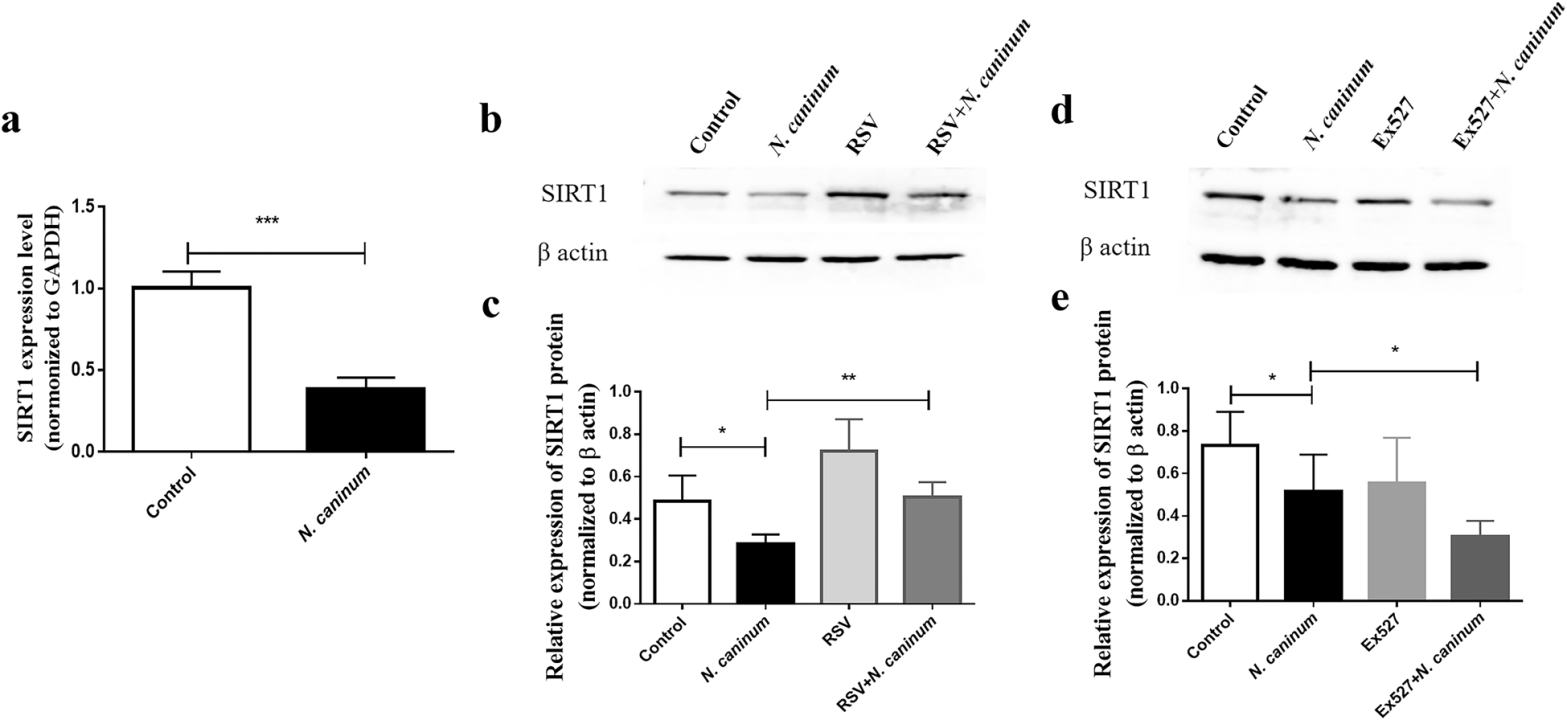
Expression analysis of Sirtuin 1 (SIRT1) in caprine endometrial epithelial cells (EECs) infected with *Neospora caninum*. **a** The mRNA levels of SIRT1 determined by RT-qPCR. The protein levels of SIRT1 determined by western blotting. RT-qPCR (**a**) and western blotting (**b**–**e**) were performed to detect the expression of SIRT1 in caprine EECs infected with *N. caninum* tachyzoites at a multiplicity of infection (MOI) of 3:1 (parasite:cell) for 48 h. Additionally, the protein levels were determined in caprine EECs pre-treated with 50 μM resveratrol (RSV) (**b**, **c**) or 20 μM Ex 527 (**d**, **e**) for 1 h and then infected with *N. caninum* tachyzoites at MOI of 3:1 (parasite:cell) for 48 h. Data are shown as mean ± standard deviation (SD) of three independent experiments. *P*-values were calculated using Student’s *t* test. *P < 0.05; **P < 0.01; ***P < 0.001

SIRT1 has been reported as an important regulator in mitochondrial biogenesis and turnover [43]. The expression of SIRT1 was investigated in caprine EECs infected with *N. caninum* for 48 h. Both mRNA (Fig. 2a) and protein (Fig. 2b, c) levels of SIRT1 were found to be significantly decreased in infected caprine EECs.

**Fig. 3:**
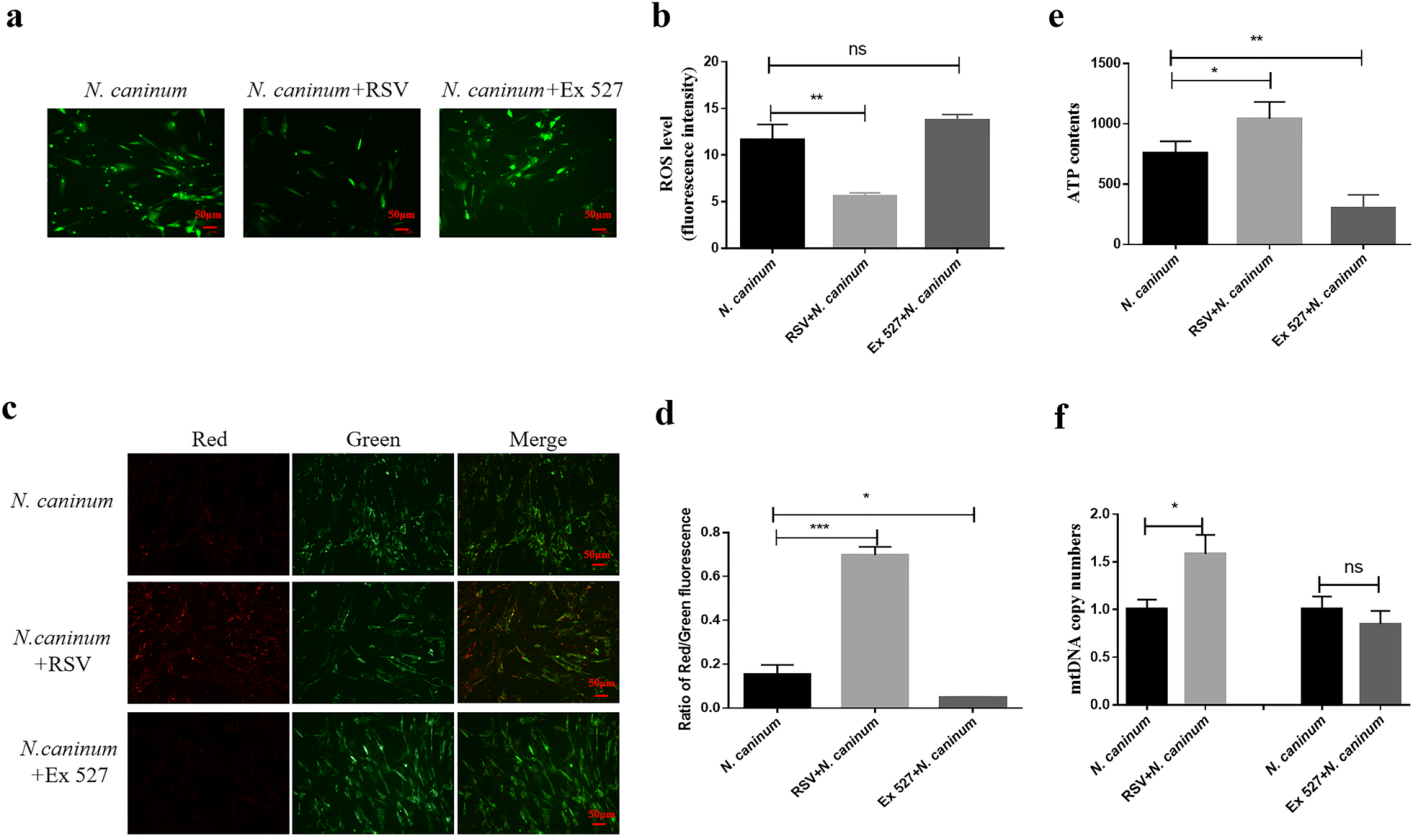
Effect of Sirtuin 1 (SIRT1) on *Neospora caninum*-induced mitochondrial dysfunction in caprine endometrial epithelial cells (EECs). **a**, **b** ROS levels detected by a DCFH-DA fluorescent probe. Scale bar, 50 µm. **c**, **d** The mitochondrial membrane potential (MMP) detected by JC-1. Scale bar, 50 µm. **e** ATP contents detected by an ATP Determination Kit. **f** Mitochondrial DNA (mtDNA) copy numbers detected by qPCR. The ROS levels, mitochondrial membrane potential, ATP levels and mtDNA copy numbers were investigated in caprine EECs pre-treated with 50 μM resveratrol (RSV) or 20 μM Ex 527 for 1 h and then infected with *N. caninum* tachyzoites at a multiplicity of infection (MOI) of 3:1 (parasite:cell) for 48 h. Data are shown as mean ± standard deviation (SD) of three independent experiments. *P*-values were calculated using Student’s *t* test. *P < 0.05; **P < 0.01; ***P < 0.001. *NS* no significant difference was observed

To determine the role of SIRT1 on mitochondrial dysfunction induced by *N. caninum* infection, two drugs, namely RSV (a SIRT1 activator) and Ex 527 (a SIRT1 inhibitor), were used to treat caprine EECs for 1 h before infection. After infection with *N. caninum* for 48 h, 50 μM RSV significantly increased the protein levels of SIRT1 induced by *N. caninum* infection (Fig. 2b, c), while the opposite results were found by using 20 μM Ex 527 (Fig. 2d, e). Interestingly, treatment with 50 μM RSV significantly reversed the effect of *N. caninum* infection on ROS levels (Fig. 3a, b), MMP (Fig. 3c, d), ATP levels (Fig. 3e) and mtDNA copy numbers (Fig. 3f) in caprine EECs, while application of 20 μM Ex 527 remarkably aggravated the impact of *N. caninum* infection on MMP (Fig. 3c, d) and ATP levels (Fig. 3e) in caprine EECs. These results indicated that *N. caninum* infection induced mitochondrial dysfunction by downregulating SIRT1 in caprine EECs.

### Effect of SIRT1 on autophagy in caprine EECs induced by *N. caninum* infection

**Fig. 4:**
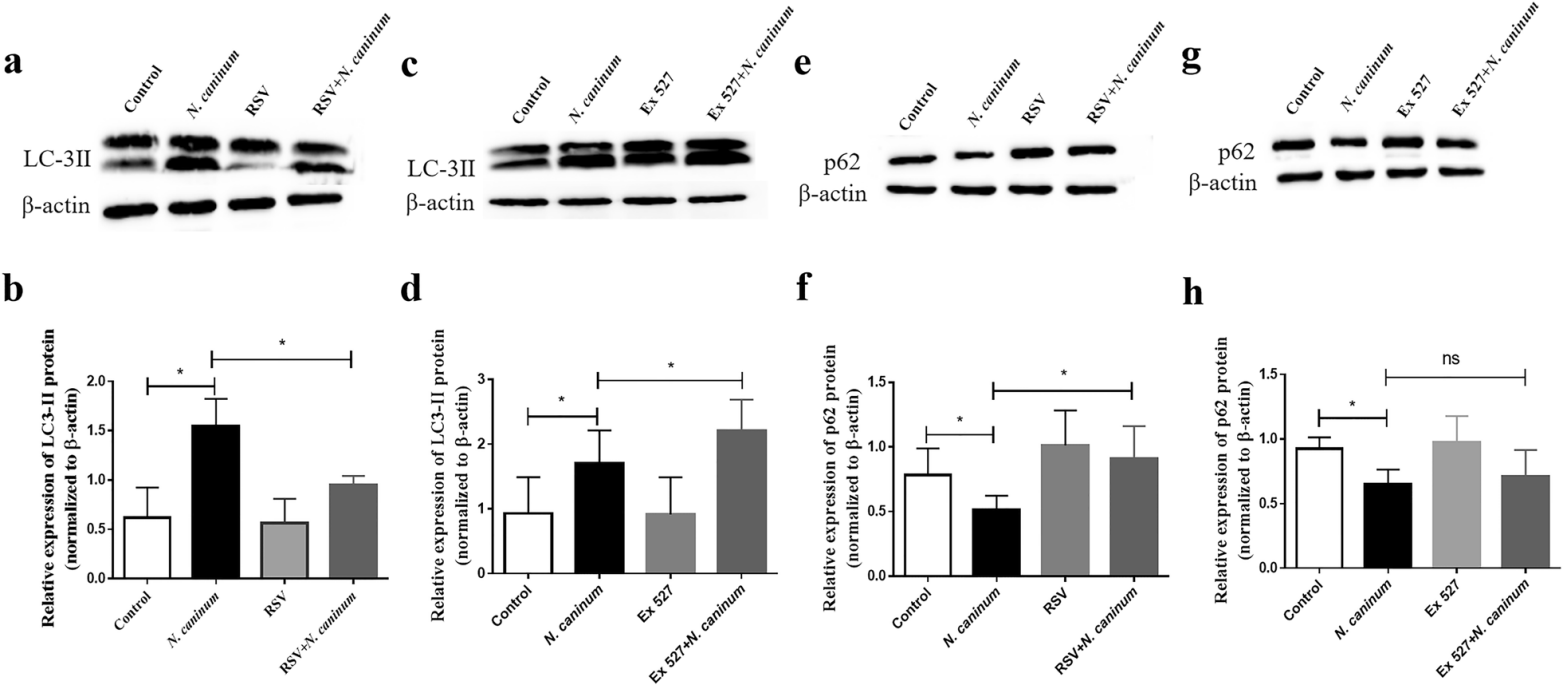
Effect of Sirtuin 1 (SIRT1) on *Neospora caninum*-induced autophagy in caprine endometrial epithelial cells (EECs). **a**–**d** The protein levels of LC-3II determined by Western blotting. **e**–**h** The protein levels of p62 determined by western blotting. Caprine EECs were pre-treated with 50 μM resveratrol (RSV) (**a**, **b**, **e**, **f**) or 20 μM Ex 527 (**c**, **d**, **g**, **h**) for 1 h and then infected with *N. caninum* tachyzoites at a multiplicity of infection (MOI) of 3:1 (parasite:cell) for 48 h. Data are shown as mean ± standard deviation (SD) of three independent experiments. *P*-values were calculated using Student’s *t* test. *P < 0.05. *NS* no significant difference was observed

Autophagy in caprine EECs induced by *N. caninum* infection was previously found by our group [41], and previous studies showed that SIRT1 was related to cell autophagy during inflammations and pathogenic infections [36, 44]. To test the effect of SIRT1 on autophagy induced by *N. caninum* infection, caprine EECs were treated with 50 μM RSV or 20 μM Ex 527 for 1 h and then infected with *N. caninum* tachyzoites for 48 h; the protein levels of LC-3II (an autophagy marker) and p62 (a molecule to monitor changes of autophagy flux) were determined. RSV treatment significantly reversed the increased expression of LC-3II and reduction of p62 caused by *N. caninum* infection (Fig. 4a, b), while Ex 527 treatment significantly increased the expression of LC-3II caused by *N. caninum* infection though had no significant effect on *N. caninum*-inducing reduction of p62 (Fig. 4c, d). These results suggested that *N. caninum* infection induced autophagy by downregulating SIRT1.

### Effect of SIRT1 on propagation of *N. caninum* in caprine EECs

**Fig. 5:**
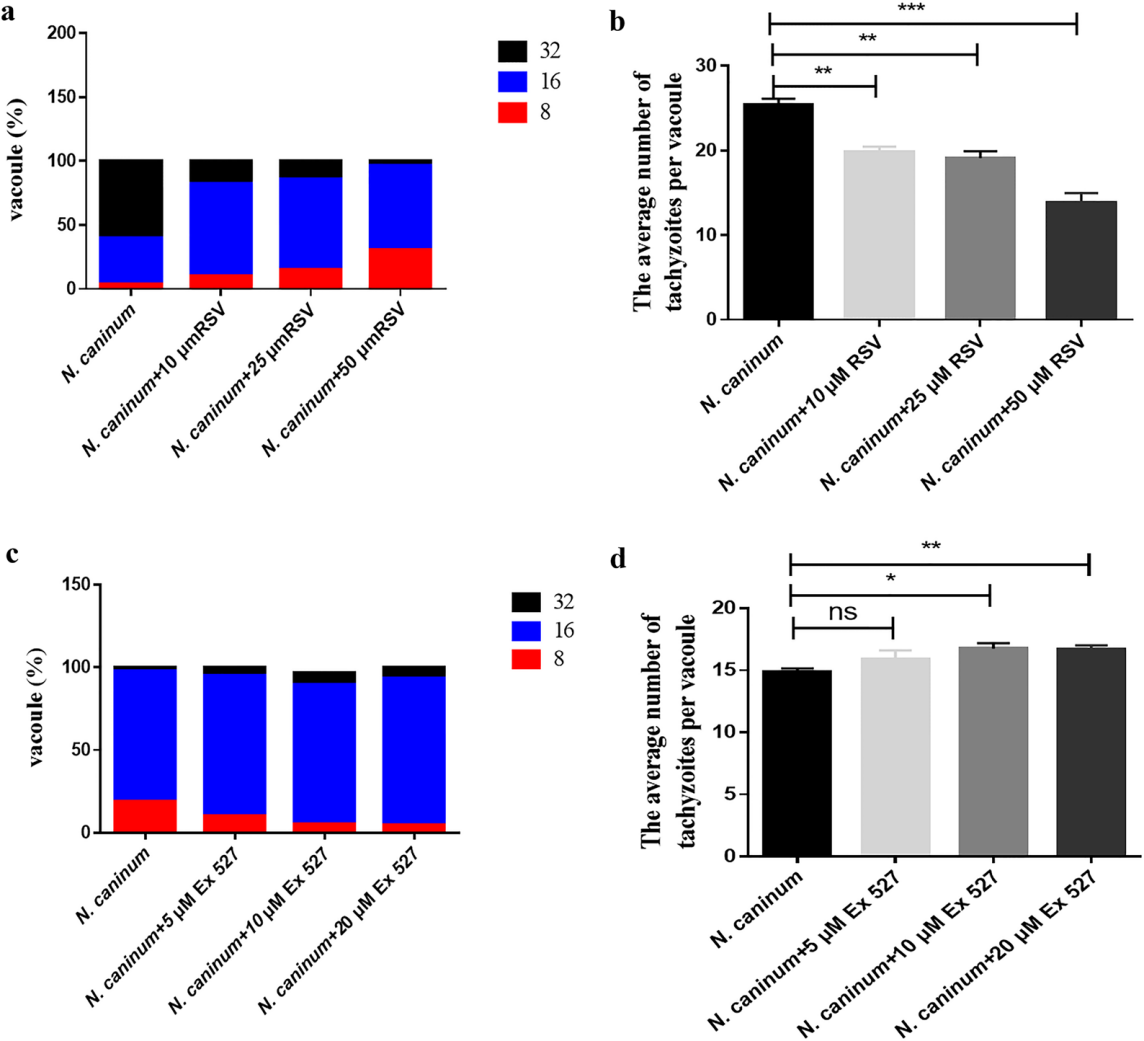
Intracellular propagation of *Neospora caninum* tachyzoites in caprine endometrial epithelial cells (EECs). Caprine EECs were pre-treated with 10, 20 and 50 μM resveratrol (RSV, **a**, **b**) or 5, 10 or 20 µM Ex 527 (**c**, **d**) for 1 h and then infected with *N. caninum* tachyzoites at a multiplicity of infection (MOI) of 3:1 (parasite:cell) for 48 h. The numbers of *N. caninum* tachyzoites of vacuoles were counted by randomly selecting 100 parasitophorous vacuoles. Three independent experiments were conducted in triplicate. Data are shown as mean ± standard deviation (SD) of three independent experiments. *P*-values were calculated using Student’s *t* test. *P < 0.05. *NS* no significant difference was observed

Autophagy induced by *N. caninum* infection has been reported to promote intracellular propagation of tachyzoites in caprine EECs by our group [41], and downregulation of SIRT1 by *N. caninum* infection advanced cell autophagy (see above). To test whether SIRT1 had a negative effect on propagation of *N. caninum* tachyzoites, caprine EECs were treated with 10–50 μM RSV or 5–20 μM Ex 527 for 1 h and then infected with *N. caninum* tachyzoites for 48 h. The average numbers of tachyzoites per parasitophorous vacuole were calculated by counting 100 vacuoles. Both RSV and Ex 527 affected replication of *N. caninum* tachyzoites in a dose-dependent manner in caprine EECs. Three dosages (10, 25 and 50 μM) of RSV significantly suppressed propagation of *N. caninum* tachyzoites in caprine EECs (Fig. 5a, b), while 10 and 20 μM of Ex 527 significantly promoted replication of tachyzoites in vitro (Fig. 5c, d). These results indicated that downregulation of SIRT1 was beneficial to propagation of *N. caninum* tachyzoites in caprine EECs.

### Discussion

Mitochondrial dysfunction has been found to be associated with many pathogenic diseases, leading to implicated outcomes, e.g. progressive cognitive decline and abortion [36, 45, 46]. Abortion is being reported as the main cause of economic losses caused by neosporosis in intermediate hosts, especially in cattle and goats [47]. Substantial evidence has suggested that mitochondrial damage was one of the main factors responsible for reproductive dysfunction [48]. Metabolism and energy demand in pregnant maternal resulted in increases in increased placental mitochondria activity and ROS generation [49]. Abnormal stimuli or external pathogenic infections also caused accumulation of mtROS and irreversible damage to mitochondria and cells (e.g. trophoblast apoptosis), finally leading to reproductive disorders [48, 50]. For example, oxidative damage-induced mitochondrial dysfunction caused by *T. gondii* would contribute to trophoblast apoptosis [51]. In the current study, accumulation of ROS was found in caprine EECs infected with intracellular *N. caninum* tachyzoites, consistent with in vivo findings in cows and gerbils [25, 52]. Notably, significant decreases of ATP contents and mtDNA copy numbers and severe disruption of mitochondrion morphology were observed in *N. caninum*-infecting caprine EECs, suggesting that mitochondrial dysfunction of endothelial cells in the uterus would be associated with pathogenesis of *N. caninum* infection.

SIRT1 has been identified to be heavily implicated in health span and longevity by controlling mitochondrial biogenesis and metabolic processes [53, 54]. Downregulation or deficiency (SIRT1^−/−^) of SIRT1 would elevate ROS production and cause ROS-induced mitochondrial function damage, enhancing pathogenesis of diseases. For example, Activation SIRT1 by using SRT1720 (an activator of SIRT1) attenuated mitochondrial dysfunction by decreasing ROS accumulation to maintain cell homeostasis in intestinal epithelial cells caused by H_2_O_2_ [55]. SIRT1^−/−^ bone marrow dendritic cell showed further decreases in MMP, ATP levels and generation of ROS during respiratory syncytial virus infection, leading to inappropriate metabolic processes and enhancement of the pathogenic responses [35]. In the current study, the expression level of SIRT1 was decreased because of *N. caninum* infection in caprine EECs. RSV treatment increased the expression of SIRT1 and reversed the effect of mitochondrial dysfunction induced by infection of *N. caninum* tachyzoites, while Ex 527 further decreased SIRT1 expression and aggravated mitochondrial damage effects caused by *N. cannum* infection. These results indicated that downregulation of SIRT1 contributed to mitochondrial dysfunction induced by infection of *N. caninum* tachyzoites and suggested a potential role of SIRT1 in pathogenesis during infection of *N. caninum*.

SIRT1 functions as both metabolic sensor and transcriptional regulator with broad cellular functions (metabolic homeostasis, stress response, tumorigenesis and autophagy) [56–59]. Of them, cell autophagy, a dynamic recycling system, has been reported to be one of common consequences due to mitochondrial dysfunction [60]. The interplay between cell autophagy and SIRT1 has been widely studied. For example, activation SIRT1 by using SRT1720 inhibited intracellular survival and colonization of *H. pylori* in gastric cells through activating autophagic flux [39]. Autophagy has been found to be induced in caprine EECs infected with *N. caninum* by downregulating mTOR, and it contributed to *N. caninum* propagation [41]. Rapamycin (an autophagy inducer) treatment increased parasite loads and reduced survival rates of *N. caninum*-infected mice [61]. Moreover, *N. caninum* infection induced mitophagy in a ROS-dependent manner to promote parasite propagation in mice and inhibited inflammatory cytokines production to achieve immune evasion [62]. This evidence suggested that autophagy/mitophagy contributed to *N. caninum* replication in vitro and in vivo. In the current study, activation of SIRT1 by using RSV inhibited autophagy induced by *N. caninum* infection, further inhibiting propagation of *N. caninum *in vitro. On the other hand, Ex 527 treatment further increased LC3-II protein expression due to *N. caninum* infection and promoted *N. caninum* replication in caprine EECs. These results indicated that *N. caninum* infection downregulated SIRT1 expression to promote autophagy and then affected propagation of *N. caninum* in caprine EECs.

Certainly, the scientific evidence has previously reported that the results obtained in studies using in vitro models were not always in accordance with the results obtained in vivo models. Considering that the abortion pathophysiology is complex, more studies are needed to understand the importance of the potential role of SIRT1 as a potential target of focus related to the treatment of *N. caninum* infection. In addition, previous work mentioned a difference in susceptibility amongst ruminant species. Therefore, it would be interesting to evaluate mitochondrial function in a model of bovine epithelial cells infected with *N. caninum* in a future study, in which *N. caninum* provokes a more severe reproductive effect in cattle. More studies are also needed on an in vivo or ex vivo model to deepen in the understanding of mitochondrial damage on the pathophysiology of abortion.

## Conclusions

The effect on mitochondrial function on caprine EECs due to infection of *N. caninum* was investigated for the first time to our knowledge. *Neospora caninum* infection downregulated SIRT1 expression to induce mitochondrial dysfunction, and downregulation of SIRT1 further promoted cell autophagy and intracellular replication of *N. caninum* tachyzoites in caprine EECs. The findings in the present study suggested a potential role of SIRT1 as a target to develop control strategies against *N. caninum* infection.

## Supplementary Information

**Additional file 1: Table S1.** Nucleotide sequences of primers used for RT-qPCR.

**Additional file 2: Figure S1.** Cytotoxic effects for resveratrol (RSV) and Ex 527 on caprine endometrial epithelial cells (EECs). Caprine EECs were seeded in 96-well cell culture plates for 24 h and then treated with 50 μM resveratrol (RSV) or 20 μM Ex 527 for 48 h. Cell viabilities were determined using a cell counting kit (CCK). Three independent experiments were performed. *NS* not statistically significant.

## Data Availability

Data supporting the conclusions of this article are included within the article.

## Abbreviations

EECs: Endometrial epithelial cells
SIRT1: Sirtuin 1
RSV: Resveratrol
ATP: Adenosine triphosphate
MMP: Mitochondrial membrane potential
ROS: Reactive oxygen species
MOI: Multiplicity of infection
JC-1: Tetrechloro-tetraethylbenzimidazol carbocyanine iodide
DCFH-DA: 2′,7′-Dichloro-fluorescin diacetate
PBS: Phosphate buffered saline
RT-qPCR: Reverse transcriptase quantitative polymerase chain reaction
TEM: Transmission electron microscopy
PVDF: Polyvinylidene fluoride
ECL: Enhanced chemiluminescence
BoHV-1: Bovine herpesvirus-1
