# Supplementary material for: Neospora caninum infection induced mitochondrial dysfunction in caprine endometrial epithelial cells via downregulating SIRT1
Source: Parasit Vectors. 2022 Aug 1;15:274. doi: 10.1186/s13071-022-05406-4 (PMC9344697; doi:10.1186/s13071-022-05406-4)
Supplement: Supplementary file 1 — Additional file 1: Table S1. Nucleotide sequences of primers used for RT-qPCR. [file 13071_2022_5406_MOESM1_ESM.docx]

Table S1. Nucleotide sequences of primers using for qRT-PCR.

| **Gene name** | **Accession number** | **primer sequence (5′–3′)** |
| --- | --- | --- |
| SIRT1 | NM_001314319.1 | F: GCTGATGAACCGCTTGCT |
|  |  | R: GCAGGTGAGGCAAAGGTTC |
| Mt DNA | X72965.1 | F：GATCCTTTCGCCTCTCTACACT |
|  |  | R：GGTCAAATGGTGCTCGGT |
| GAPDH | XM_005680968.3 | F：TCCACGGCACAGTCAAGG |
|  |  | R：TCAGCACCAGCATCACCC |
| 18s rRNA | DQ149973.1 | F：CCAGTAAGTGCGGGTCAT |
|  |  | R：CCATCCAATCGGTAGTAGCG |
