# Supplementary figures and images for: Neospora caninum infection induced mitochondrial dysfunction in caprine endometrial epithelial cells via downregulating SIRT1
Source: Parasit Vectors. 2022 Aug 1;15:274. doi: 10.1186/s13071-022-05406-4 (PMC9344697; doi:10.1186/s13071-022-05406-4)

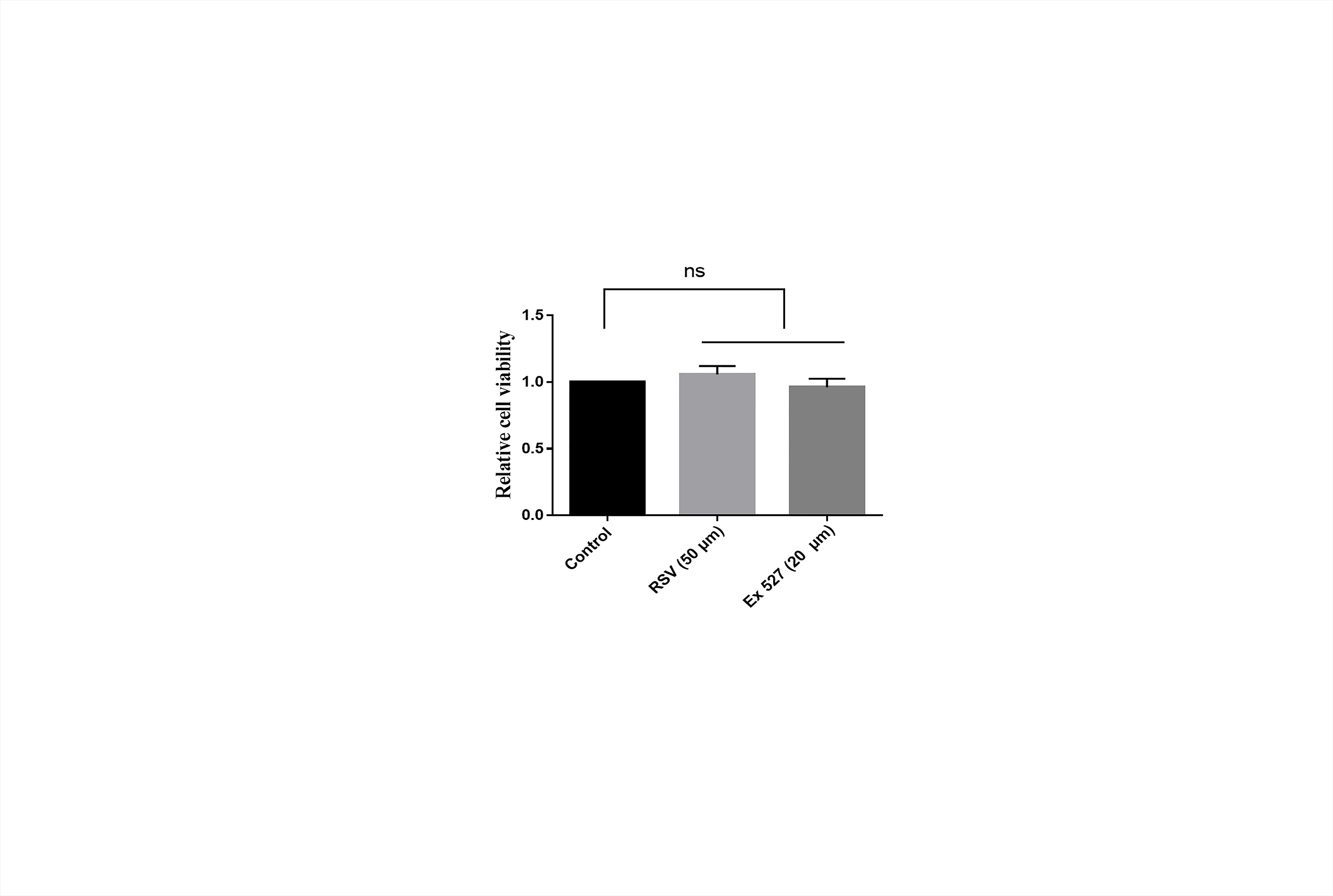

Supplement: Supplementary file 2 — Additional file 2: Figure S1. Cytotoxic effects for resveratrol (RSV) and Ex 527 on caprine endometrial epithelial cells (EECs). Caprine EECs were seeded in 96-well cell culture plates for 24 h and then treated with 50 μM resveratrol (RSV) or 20 μM Ex 527 for 48 h. Cell viabilities were determined using a cell counting kit (CCK). Three independent experiments were performed. NS not statistically significant. [file 13071_2022_5406_MOESM2_ESM.tif]
